# NightHawk: A Low-Cost, Nighttime Light Wildfire Observation Platform and Its Radiometric Calibration

**DOI:** 10.3390/s25072049

**Published:** 2025-03-25

**Authors:** Chase A. Fuller, Steve Tammes, Philip Kaaret, Jun Wang, Carlton H. Richey, Marc Linderman, Emmett J. Ientilucci, Thomas Schnell, William Julstrom, Jarret McElrath, Will Meiners, Jack Kelley, Francis Mawanda

**Affiliations:** 1 Department of Physics & Astronomy, The University of Iowa, Iowa City, IA 52242, USA; philip.kaaret@nasa.gov (P.K.); jun-wang-1@uiowa.edu (J.W.); jarret-mcelrath@uiowa.edu (J.M.); william-meiners@uiowa.edu (W.M.); jack-e-kelley@uiowa.edu (J.K.);; 2 Department of Chemical and Biochemical Engineering, The University of Iowa, Iowa City, IA 52242, USA; steven-tammes@uiowa.edu (S.T.); william-julstrom@uiowa.edu (W.J.); 3 NASA Marshall Space Flight Center, Huntsville, AL 35812, USA; 4 Operator Performance Laboratory, Iowa City, IA 52246, USA; carlton-richey@uiowa.edu (C.H.R.); thomas-schnell@uiowa.edu (T.S.); 5 Department of Geographical & Sustainability Sciences, The University of Iowa, Iowa City, IA 52242, USA; marc-linderman@uiowa.edu; 6 Chester F. Carlson Center for Imaging Science, Rochester Institute of Technology, Rochester, NY 14623, USA; emmett@cis.rit.edu

**Keywords:** radiometric calibration, fire detection, wildfire, nighttime fire activity, flaming, nighttime lights, multiband imaging, aircraft remote sensing

## Abstract

We present a low-cost prototype of a visible and near-infrared (VIS-NIR) remote sensing platform, optimized to detect and characterize natural flaming fire fronts from airborne nighttime light (NTL) observations, and its radiometric calibration. It uses commercially available CMOS sensor cameras and filters with roughly 100 nm bandwidths to effectively discriminate burning biomass from other sources of NTL, a critical ability for wildfire monitoring near populated areas. Our filter choice takes advantage of the strong potassium line emission near 770 nm present in natural flaming. The calibrated cameras operate at 20 ms of exposure time and boast radiance measurements with a sensitivity floor, depending on the filter, in the range 3–5 $\times \;10^{-6}$ W m^−2^ sr^−1^ nm^−1^ with uncertainties lower than 5% and dynamic ranges near 3000–4000. An additional exposure time with a tenth of the duration is calibrated and extends the dynamic range by a factor of 10. We show images of a spatially resolved fire front from an airborne observation of flaming biomass within this radiance range.

## 1. Introduction

Wildfires can be both costly and deadly when they become out of control near populated areas. Even when wildfires do not threaten human life, they generate large quantities of particulate matter and greenhouse gases, both of which have far-reaching effects on Earth’s atmosphere and public health once released [1]. As such, it is critical to locate wildfires as they begin, estimate their severity, and monitor their time evolution. Autonomous and semi-autonomous remote sensing provides a way of monitoring large swaths of Earth’s surface for fire activity [2].

There are several ways that a fire can be remotely sensed. Typical observation strategies include short-wave infrared (SWIR) and mid-wave infrared (MWIR) sensors, operating at wavelengths of λ = 0.9–2.5 μm and λ = 3–5 μm, respectively. Techniques to measure fire radiative power (FRP) using infrared imagers also have a proven track record (e.g., [2,3]). Beyond that, imagers that operate at longer wavelengths in the thermal infrared are also used. Each of these bands are advantageous because fires tend to exhibit high radiance in the infrared, and the atmosphere and fire smoke are mostly transparent at long wavelengths. MWIR tends to be the band of choice, because contamination from sunlight is stronger in the SWIR. Infrared sensors, unfortunately, tend to suffer from high cost and are sensitive to their operating temperature. Infrared photons have lower energy than their visible counterparts, so high-resolution IR imaging semiconductor devices must have small enough bandgaps to be sensitive to these low-energy photons. These sensors generally require a robust cooling solution to reduce contamination from thermal electrons entering the sensor’s conduction band.

The Visible Infrared Imaging Radiometer Suite (VIIRS) instruments operating aboard Suomi-NPP and the NOAA-20 satellites offer fire detection from space using multispectral observations with a ground resolution of 375 m [4]. Recent VIIRS algorithm improvements, specifically with the Fire Light Detection Algorithm (FILDA-2), have increased the quality of VIIRS fire data products, including measurements of the visible emission fraction (VEF) [5]. The technique uses radiance measurements from VIIRS’s day/night band (DNB), operating in the visible and near-infrared (VIS-NIR), and radiances measured by the M13 band, operating in the MWIR. VEF, the ratio of visible light radiance emitted to infrared light radiance, may be a better indication of fire propagation direction than the fire radiative power alone [5]. For example, space-based FRP measurements lack the spatial resolution to discriminate between a pixel dominated by a large area of smoldering, or by a small area of active flaming—which might have equal estimates of FRP. The small area of active flaming, however, is much more likely to propagate the fire front, since a fire front occurs at the boundary between burned and unburned fuel.

The VEF has also been found to be related to the fires’ modified combustion efficiency (MCE) [5,6]. The MCE of wildfires, the mass ratio of carbon emitted as CO_2_ to the total carbon emitted as both carbon dioxide and carbon monoxide, is intimately related to the diversity and quantity of other chemical species released by burning biomass. Very efficient combustion of burning biomass releases mostly carbon dioxide, water, and heat. Emission of other fire byproducts, like gaseous oxides of nitrogen and sulfur, volatile, semi-volatile, and non-volatile organic compounds, as well as aerosolized particulate matter are minimized when combustion efficiency is high. Conversely, when combustion is inefficient, large quantities of the other fire byproducts are released [7]. These pollutants have far-reaching effects on human health [7,8] and the atmosphere [9]. Burning biomass aerosols and chemical species are also each important inputs to climate forecasting models [10]. Measuring a fire event’s combustion efficiency is critical to forecast the overall impact of a fire event. With that in mind, we are constructing a remote sensing platform optimized to measure the VEF, and therefore the modified combustion efficiency, of burning biomass. Given that proven techniques exist to measure FRP in the MWIR, this paper is dedicated to the prototyping of the VIS-NIR half of the array of sensors required to measure the VEF.

Operating in the VIS-NIR allows us to use silicon-based semiconductor cameras. High-resolution, commercialized complementary metal–oxide–semiconductor (CMOS) sensors are relatively insensitive to operating temperature and are inexpensive. While they lack sensitivity deeper into the infrared spectrum, they are sensitive in the VIS-NIR. Burning biomass flaming is bright in the VIS-NIR, especially due to an excited potassium line emission near 770 nm. Natural flaming, from a fire whose fuel is plant matter, typically has a large quantity of excited potassium since plants draw potassium from the ground and sequester it within their biomass. Burning this biomass excites a disproportionate amount of potassium line emission compared to other kinds of flaming. Using an overabundant potassium line emission from observed light sources to identify flaming was suggested by Vodacek et al. [11] and investigated for use in daytime fire detection [12]. Vodacek et al. [11] suggest using a K-emission index—the ratio of narrow band observations near 770 nm to a nearby band that does not contain the potassium emission, e.g., 780 nm. Unfortunately, near-infrared observations of excited potassium emission during the day are fraught with background contamination. Sunlight is bright near 770 nm, and vegetation is reflective in the near-infrared [13].

NightHawk’s observation strategy circumvents the daytime contamination problem entirely by focusing on observing nighttime light. This is not only a matter of observation convenience, Balch et al. [14] showed that wildfire activity at night has increased across the globe. A platform optimized for observing nighttime wildfire activity, especially active flaming, could prove to be a valuable tool. We investigated whether or not burning biomass could be detected using commercially available CMOS sensors and broadband VIS-NIR filters in a previous publication [15]. In that communication, we showed that it is indeed possible to discriminate between burning biomass and other sources of NTL using accessible hardware and a simple band-ratio technique. The ability to distinguish between flaming and artificial light is crucial, as wildfires pose the greatest immediate danger when they approach populated areas, which are typically well lit by artificial lights.

While the uncalibrated data from NightHawk proved to be useful for discriminating between light from burning biomass and artificial nighttime light, here we conducted a radiometric calibration of NightHawk’s VIS-NIR sensors. Radiometrically calibrating our four VIS-NIR cameras provides sensors that can be used to detect and characterize active flaming from burning biomass. In particular, absolutely calibrated radiance measurements of light in the VIS-NIR are required to measure a fire’s visible emission fraction. In the following sections, we present the NightHawk device, discuss its radiometric calibration, and show calibrated measurements of a spatially resolved fire front.

## 2. NightHawk

### 2.1. Construction and Operation

**Figure 1:**
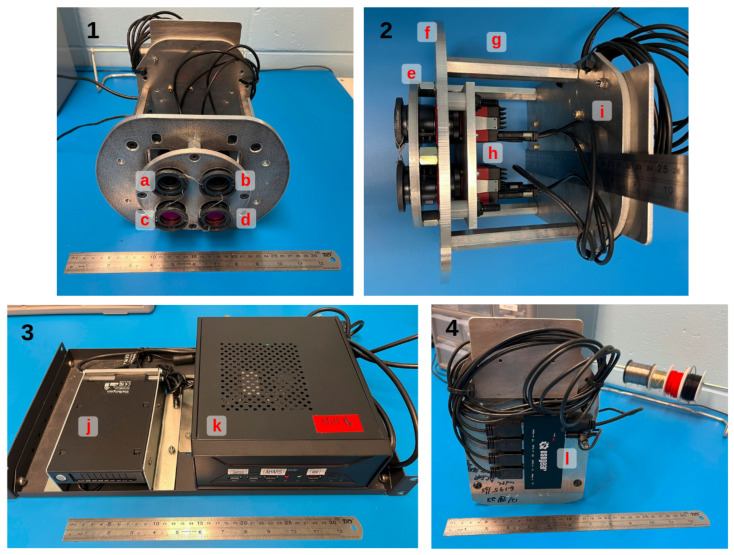
NightHawk panels (**1**,**2**,**4**) and the XH410G computer panel (**3**) that runs it. A ruler is included in each image for a sense of scale. Panel 1 shows each of the 4 cameras labeled by their MidOpt filter: (a) BP735, (b) BP470, (c) BP635, and (d) BP540. The BP635 and BP540 cameras have a second, external SP700 filter that is IR-reflecting. The camera lenses are safety-wired together using modified step-rings and stainless-steel wire. A view from the side is shown in panel 2 that highlights the quarter-inch machined aluminum plates and the cameras. The cover plate (e) fills the 5” diameter port of the aircraft. The base plate (f) mounts the device to the aircraft. Stand-offs (g) separate the base plate and the top plate (i), which protects the rear of the cameras and mounts the powered USB hub (l) shown in panel 4. Item (h) is a side view of the Alvium cameras themselves and the camera plate they are mounted to. Panel 3 shows the external, removable 2.5” solid-state drive bay (j) where data are recorded and transferred to a server later. Item (k) is the computer that runs the device.

This iteration of NightHawk was designed and built to be deployed on a Beechcraft Bonanza aircraft. The aircraft we used has been retrofitted by our team members at the Operator Performance Laboratory (OPL) for scientific applications. The four cameras are arranged in a 2 × 2 pattern to fit within a 5-inch-diameter port on the bottom of the aircraft. The cameras are connected to a powered USB hub and the hub is connected to a computer operated by an experimenter in the aircraft. We use Allied Vision’s software development kit (SDK) Vimba 6.0 to control the camera system run by a ruggedized computer also mounted to the aircraft. NightHawk and its computer are shown in Figure 1. The software’s image acquisition cadence acquires one image with each camera at 20 ms of exposure time and two additional images with the red and NIR cameras at 2 ms of exposure time.

NightHawk’s current mechanical design, shown in Figure 1, is optimized for the platform it is deployed on. Most of the constraints on the design are based purely on the dimensions of the aircraft’s mount and view port. The device is made primarily out of machined aluminum plates 1/4 of an inch thick. The base plate that mounts to the aircraft is the largest and supports the rest of the device. A separate, contiguous plate holds the cameras and the inertial measurement unit (IMU) used to track the cameras’ position and attitude information. We chose to use a single plate to hold all sensors because this produces the most stable optical alignment. The plate holding all of these sensors is separated from the base plate by nylon screws and standoffs long enough to keep the front of the camera lenses close to flush with the bottom of the aircraft. The lenses are safety-wired compliant with Federal Aviation Administration standards to prevent them from becoming loose during operation. A cover plate fills the gap between the cameras and covers the majority of the port on the aircraft. Above the cameras is a final plate that both protects the rear of the cameras and holds the USB hub.

### 2.2. Sensors

**Figure 2:**
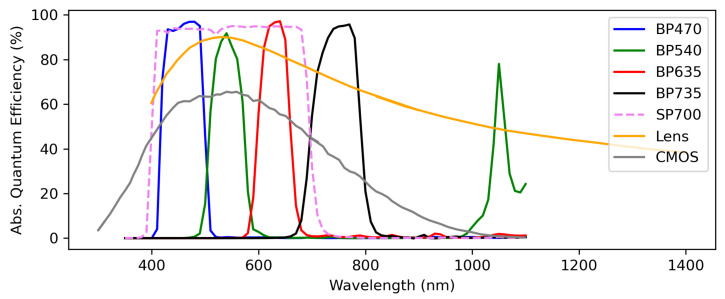
The absolute quantum efficiencies and transmissions of the various optical components provided by their respective manufacturers. Note that the BP635 and BP540 filters exhibit out-of-band transmission at long wavelengths, which is eliminated by pairing them with the SP700 filter. The CMOS sensor also loses sensitivity near 1000 nm, which doubly accounts for the BP540 filter’s extraneous infrared transmission.

NightHawk employs 4 monochromatic Alvium U-1800 319m cameras manufactured by Allied Vision (Stadtroda, Germany). These cameras use IMX265 complementary metal–oxide–semiconductor (CMOS) sensors manufactured by Sony (Tokyo, Japan). They are in a 2/3 of an inch format, with 1544 × 2064 pixels. They have reasonable quantum efficiency in the visible and near-infrared, peaking at 65% near 529 nm, in comparison with other sensors of similar cost. The absolute quantum efficiency of the sensor is shown in Figure 2. The 319m cameras are also capable of recording images with 12 bits of precision. More details of this camera configuration are available in Allied Vision’s EMVA1288 compliant report [16].

The 4 cameras are outfitted with 6 filters made by Midwest Optical Systems, or MidOpt (Palatine, IL, USA). The filter bandpasses represent the blue (BP470), green (BP540), red (BP635), and NIR (BP735) parts of the VIS-NIR spectrum. The width of the bandpasses can be seen in Figure 2. The green and red filters are paired with an additional infrared-reflective SP700 filter to eliminate undesired transmission at long wavelengths. The red, green, and blue filters were chosen to complement the NIR filter. The NIR filter was chosen specifically because it contains a wavelength range where (1) we expect to find excited potassium line emission to meaningfully contribute while also not extending the bandpass further into the infrared, and (2) the IMX265 CMOS sensor is still reasonably sensitive. By taking band ratios in the blue/green and the NIR/red, we can discriminate between burning biomass and unnatural sources of NTL. We can also subclassify artificial lights into different groups (fluorescent, LED, incandescent, etc.). The light source discriminatory power of this filter suite has been demonstrated in a previous publication [15]. The burning biomass signal in the near infrared is enhanced in the BP735 image relative to other light sources due to the contribution of excited potassium line emission in the BP735 passband.

For lenses, we selected the LM8JCM-V model manufactured by Kowa (Nagoya, Japan). The LM8JCM-V is ruggedized and vibration resistant, which are each critical features for our use case. With the 2/3” format of the IMX265 sensor, these lenses offer a horizontal × vertical × diagonal FOV of $56.5^{\circ }\times 43.9^{\circ }\times 67^{\circ }$ in diameter. In addition to the lenses working well within our size, weight, and vibration constraints, Kowa also supplies a measured quantum efficiency curve for this lens model, presented in Figure 2. The measured quantum efficiency curve is a necessary component in calculating a theoretical spectral response of each camera, which we compare later with measured relative spectral responses (RSRs) in Section 3.4.

The last sensor is Movella’s Xsens MTI-G-710-2A8G4 IMU (Henderson, NV, USA). It is mounted to the same plate as the cameras, to monitor the position, velocity, acceleration, and pointing direction of the device in real time. These ancillary data are necessary to georectify the images gathered by NightHawk.

### 2.3. Dark-Level Performance

We quantify the dark-level performance of our cameras by examining the average behavior of 100 dark frames. We set a dark-level bias to allow statistical fluctuations in the pixels to report digital numbers *N* above and below the set bias level. Without a bias, an electronic fluctuation that would result in a negative digital number would truncate to N=0, which would prevent us from sampling the expected normal distribution of the dark-level behavior. The standard deviation divided by the average pixel value in the averaged dark frame is about 0.35%. We can account for some of the variation in our images by subtracting this mean dark frame from the rest of the data since we fix the bias. This is important because in NTL observations there may be many regions in a single image that have no illumination at all, in which case the pixels in the dark region approximate the dark level of the whole image.

Sets of dark frames also allow us to find hot pixels, which are pixels that systematically report digital numbers that are unreasonably high and typically exist due to a manufacturing fault. We conservatively set the hot-pixel threshold to be pixels that report digital numbers greater than three standard deviations above the mean in an average dark frame. Hot pixels found this way can then be neglected in subsequent analyses.

## 3. Radiometric Calibration

A radiometrically calibrated camera reports radiance instead of digital numbers. We seek a function that links measured digital numbers to the incident radiance responsible for producing them. Since our system has a response that is wavelength dependent, a known radiance is insufficient. The light source must have a known spectral radiance. That is, a light source that has a well characterized power output per emitting area per solid angle per wavelength. The light source should be uniform (commonly referred to as flat) and large enough to fill the sensor’s field of view. The light source intensity should be adjustable, such that the dynamic range of the sensors can be probed. The spectral radiances at each intensity level are then folded through the sensor response to obtain a sensed or effective radiance. Conversions coefficients are found by performing a linear regression on effective radiance versus digital number.

The sensor’s relative spectral response (RSR) can either be measured using a monochromator or calculated. A simple calculation involves taking the product of all of the relevant optical components’ transmission and detection efficiencies as a function of wavelength. For NightHawk, we need the absolute spectral transmission efficiency of the camera lens (also referred to as the lens’s quantum efficiency), the filter transmission curves, and the absolute quantum efficiency of the camera’s silicon sensor itself. If it is to be measured, then we require a monochromator that produces known power outputs at known wavelengths. We then increment the monochromator wavelength to scan across the sensitive range of the system.

Our calibration procedure is outlined in the following sections. We performed the radiometric calibration of NightHawk at the Optical Calibration Facility (OCF) at the Rochester Institute of Technology (RIT). We used their LabSphere (North Sutton, NH, USA) HELIOS 20-inch-diameter integrating sphere and attached an absolutely calibrated spectrometer, their two plasma light sources shining into the sphere, and both their Newport (Stratford, CT, USA) 130 1/8-meter monochromator with 3.7 nm wavelength spacing and their Optronics Labs (Orlando, FL, USA) OL750 monochromator.

### 3.1. Sensor Model

The digital numbers that a camera reports are proportional to the amount of light sensed by the camera sensor. The physical quantity that produces a response from the camera is the incident spectral radiance $L_{\lambda }$ (in units of W m^−2^ sr^−1^ nm^−1^) from some object in the camera’s field of view. Note that the sensor does not measure the incident spectral radiance itself—the camera optics alter the incident light and the silicon sensor has a detection efficiency dependent on wavelength. For example, a filter will truncate an arbitrary incident spectral radiance to only the wavelengths the filter transmits. Instead, the instrument measures an effective radiance $L_{\mathrm{eff}}$ which depends on the camera system’s particular response to an incoming spectral radiance $L_{\lambda }$. The effective radiance $L_{\mathrm{eff}}$ is computed from the spectral radiance $L_{\lambda }$:

(1) $$L_{\mathrm{eff}}=\frac{\int_{\lambda_{\min}}^{\lambda_{\max}}{\mathrm{RSR}}_{\lambda }\cdot L_{\lambda }d\lambda }{\int_{\lambda_{\min}}^{\lambda_{\max}}{\mathrm{RSR}}_{\lambda }d\lambda },$$

where ${\mathrm{RSR}}_{\lambda }$ is the system’s peak-normalized relative spectral response (RSR) (e.g., [17]). Measuring the RSR quantifies the transmission and detection efficiencies of the sensor and optics and allows us to calculate an effective radiance from an arbitrary incident spectral radiance. The next section provides the details of measuring each of our sensor’s RSRs.

The relationship between digital number *N* and effective radiance $L_{\mathrm{eff}}$ is simple in the optical regime:

(2) $$L_{\mathrm{eff}}=G\left(N-D\right),$$

where *D*, in units of ADU, is an offset associated with the dark level of the instrument and *G* is the calibration coefficient (in units of ADU^−1^ W m^−2^ sr^−1^ nm^−1^). Our objective is to determine both *G* and *D*. To accomplish this task, we need several measurements of $L_{\mathrm{eff}}$ at different light levels and their corresponding *N*. In order to obtain values for $L_{\mathrm{eff}}$, however, we need measured RSRs.

### 3.2. Flat Fielding

It should be noted that the digital number recorded by a camera is also a function of other things in addition to the dark level and incident radiance. Gain, exposure time, and vignetting must, in general, be accounted for. If operational parameters like gain and exposure time can be fixed, then performing a calibration is simplified. Variable exposure times and gains require a more sophisticated treatment; e.g., [18,19]. The dark levels of the cameras were measured by taking dark frames and should correspond to the intercept *D* in Equation (2), unless the average dark behavior is subtracted from the calibration data. If the dark level is subtracted, then the intercept should be consistent with zero. A vignette filter is measured by taking flat-field images, subtracting the dark level from the mean of the flat-field images and normalizing the result to the pixel value at the optical axis. For us, this is the value of the pixel at the center of the circularly symmetric light pattern of the light focused on the CMOS sensor itself.

**Figure 3:**
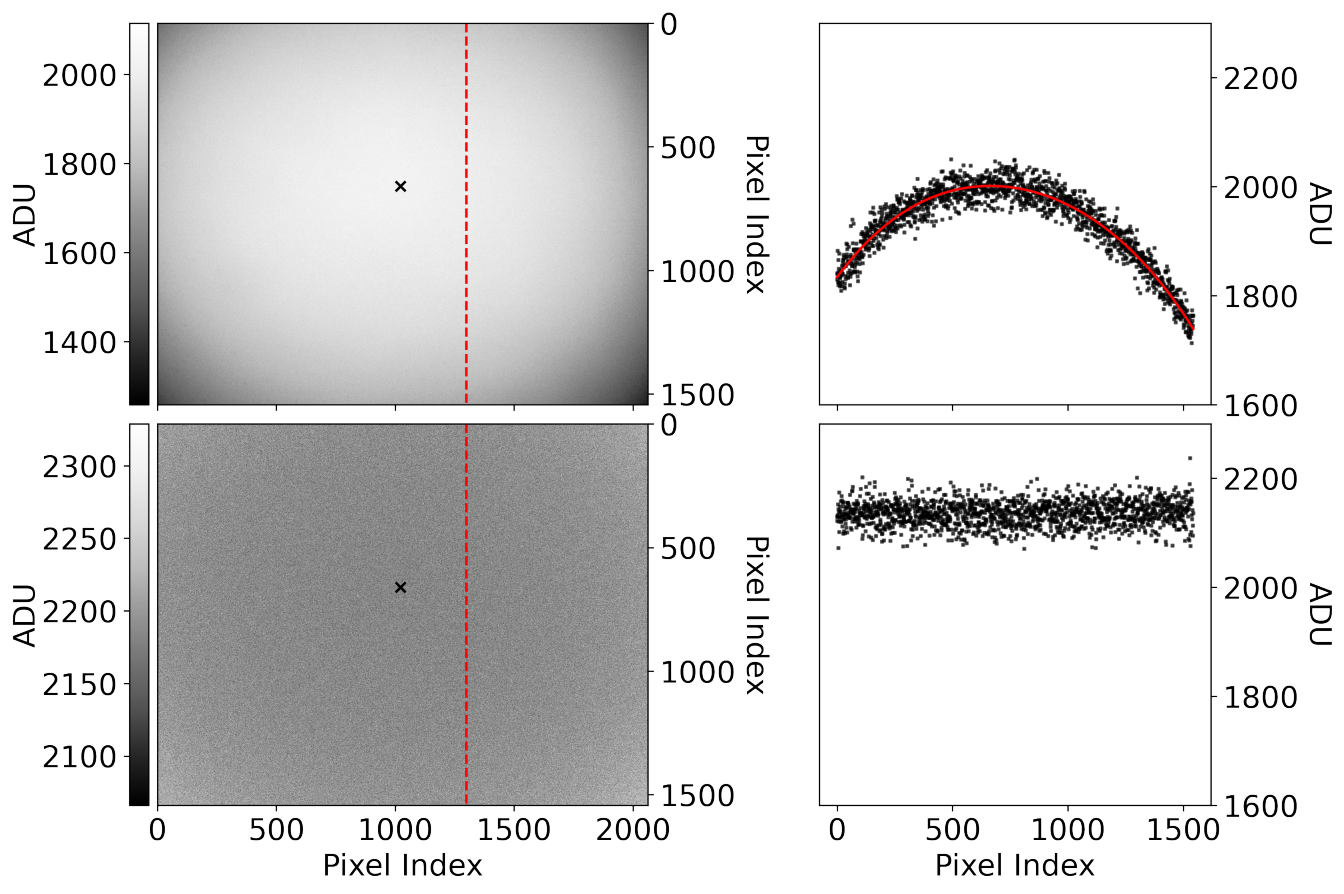
(**Top left**): An average flat field with its optical center, found according to the text, shown as an ‘x’. The vertical red dashed line is a column of data shown in black in the top right plot. (**Top right**): An example polynomial fit (red) to a column of data (black) in the average flat field. Note that the location of the maximum digital count does not appear to coincide with the location of the maximum value of the fit and that the shape appears offset. (**Bottom left**): A flat-field image corrected by dividing it by the average flat field normalized to the pixel value at its optical center. (**Bottom right**): Pixel values in the corrected image from the column of pixels shown as a dashed red line in the bottom left panel. The corrected image has no remaining vignette.

There is some natural pixel-to-pixel variation in every image due to detection statistics. In every image, the pixel value that should ideally be recorded may not be the one that is actually recorded. The statistical fluctuations in the flat field impact the location of the optical axis. In a world without statistical fluctuations, the reported digital number at the optical axis would be the maximum digital number in the image. We find the optical axis by fitting polynomials to the rows and columns of the image; e.g., Figure 3. Fitting polynomials to the rows and columns of the image produces a smoothed vignette map that is insensitive to the pixel-to-pixel variations and contaminating features that can make the location of the optical axis ambiguous. In the smooth vignette map, the location of its maximum, and therefore the location of the optical axis, is unambiguous. We normalize the mean flat-field image to its pixel value at this location to obtain a vignette filter. Subsequent frames can then be corrected by dividing by this vignette filter (an example of this is also shown in Figure 3).

### 3.3. CMOS Measurement Uncertainty

**Figure 4:**
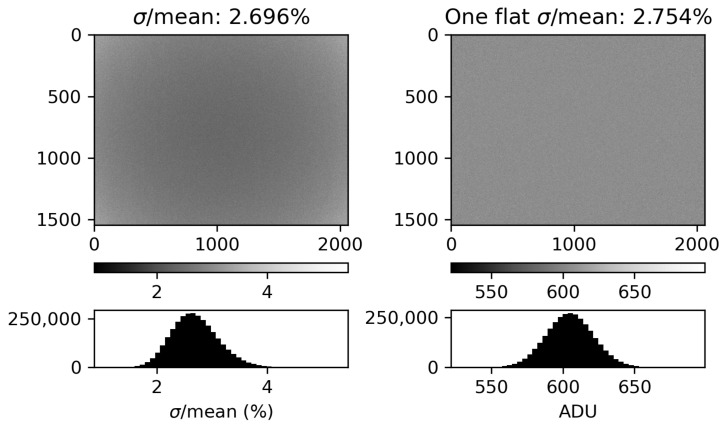
(**Left**): A 2D matrix whose elements correspond to individual pixels’ standard deviation over the mean value according to 100 measurements of the same external stimulus, thus quantifying the distribution of digital numbers each pixel records. On average, the uncertainty is 2.696%. (**Right**): A single flat field. The standard deviation of the corrected digital numbers over the average digital number is 2.754%. Note that in both cases the data are dark-subtracted and vignette-corrected. The uncertainty here compares well with the 2.5% we expect from shot noise alone.

We examined whether or not we could assume the behavior of each pixel in the sensor is statistically the same. If this is the case, then we can use the digital numbers recorded in an individual flat frame as if they are sampling the statistical behavior of each individual pixel. To examine the consistency of the pixel response to an incident radiance, we exposed the camera to a uniform source of illuminance and acquired 100 flat frames. We corrected the flat frames by first subtracting a mean dark frame and then, for vignetting, by dividing by a vignette filter acquired according to the previous subsection. This dataset provides 100 samples of how each pixel responds to the same external stimulus. The average of the standard deviation over the mean of these 100 samples at each pixel, 2.70%, compares well with the bulk behavior of a single flat frame. Neglecting hot pixels, the standard deviation of the pixel values in a single flat frame is σ/mean=2.75%. Figure 4 shows the results of this analysis with the 100-sample standard deviation over the mean on the left and the individual flat frame on the right. From here, we can assume (1) each pixel behaves the same and (2) if the digital number is sufficiently large (i.e., the number of samples from the Poissonian probability of detection is sufficiently large), the associated measurement uncertainty at each pixel is 2.7%.

We now compare the measurement uncertainty result from the empirical discussion above with the theoretical, ideal measurement uncertainty due to shot noise. The production of photoelectrons $e^{-}$ from photons incident on the CMOS sensor is Poissonian in nature—in other words, each measurement samples from a Poisson distribution. Conveniently, the standard deviation of a Poisson distribution is equal to the square root of its average. Assuming the number of samples from the camera’s Poissonian detection statistics is sufficiently large (the reported digital number is large enough), we can use this fact to calculate the σ/mean we measured empirically in Figure 4.

We use the data in the right panel of Figure 4. The mean digital number is $\langle N\rangle =604.8$ ADU. The gain of the cameras *g* is measured and reported in the EMVA 1288 document as $g=2.653\;e^{-}\cdot {\mathrm{ADU}}^{-1}$ [16]. The mean number of photoelectrons is then $\langle n\rangle =g\cdot \langle N\rangle \;\left(e^{-}\right)$. We compute $\sqrt{\langle n\rangle }/\langle n\rangle =2.5\%$, which compares well with our empirical measurement of 2.7%.

In summary, we compared individual pixel behavior with the bulk behavior of a whole image. There does not appear to be any significant contribution from pixel-to-pixel variations, and Poisson statistics dominates the measurement uncertainty of our sensor. This implies that every pixel on the camera behaves similarly and allows us to use one image to produce a high-fidelity radiance measurement during the calibration procedure.

### 3.4. RSR Measurement

We used a Newport monochromator to measure the relative spectral response (RSR) of our sensors. The RSR tells us how a sensor ‘sees’ some arbitrary incident spectral radiance. Each optical component in a sensor system alters light as the light passes through it. For example, a filter only allows light to transmit at particular wavelengths, and even a transparent lens will affect light as it transmits. For us, each RSR is dominated by the chosen filters. Each RSR should have a full width at half maximum (FWHM) of about 100 nm for the four sensors. The monochromator we used allowed us to probe the relative spectral response of our sensors at each wavelength step of the monochromator. Since our filters’ FWHMs are relatively wide, the 3.7 nm wavelength spacing of the monochromator we used was fine enough to accurately measure the shape of each RSR.

**Figure 5:**
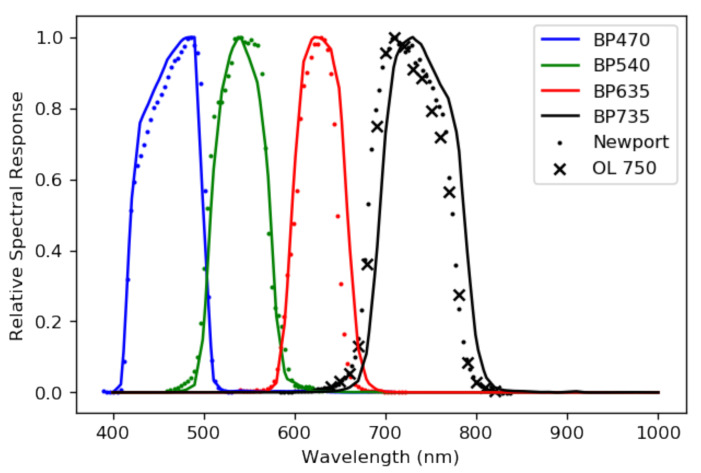
The calculated RSRs (solid lines) and their measured counterparts (dots). There appears to be good agreement between the calculated and measured RSRs for the BP470, BP540, and BP635 cameras. There is a clear discrepancy between the calculated BP735 RSR and the measured one. We verified the apparent horizontal translation of the RSR by taking additional monochromator measurements with a second monochromator (crosses). The discrepancy is within the ±10 nm margin afforded by the manufacturer.

The cameras, focused to infinity, were placed as near to the viewing port of the monochromator as was possible. The signal from the port did not fill the FOV of our cameras, so we selected a region of the image from which to extract the camera response as a function of wavelength. An appropriate region is a region of the data where the view port of the monochromator is in view. There were some minor systematic contaminants, like reflections from the edge of the viewing port, that prevent us from simply choosing to incorporate data from the pixels with a signal. We used a Gaussian blur on the data collected to wash out the effects of small contaminants and programatically find the center of the monochromator view port. The digital numbers *N* recorded by the cameras at each wavelength from the monochromator were normalized by the known power output of the monochromator at each wavelength step, which produced an RSR with units of ADU W^−1^. The RSR was then peak-normalized. The measured, peak-normalized RSRs are shown in Figure 5.

Given the relative simplicity of our system’s optics, it is a worthwhile exercise to compare the measured RSRs with those computed from the relevant transmission and detection efficiencies, also shown in Figure 5. We estimated the sensor spectral response $R_{\lambda }$ using the equation

(3) $$R_{\lambda }=\tau_{\mathrm{lens}}\times \mathrm{QE}\times \tau_{\mathrm{filter}},$$

where $\tau_{\mathrm{lens}}$ and QE are the spectral transmission of the lens and CMOS sensor spectral quantum efficiency, respectively. $\tau_{\mathrm{filter}}$ is the filter spectral transmission, and in the case where there are two filters, it is the product of both of the filters’ transmissions. Taking the above product and peak-normalizing the results gives the estimated theoretical responses plotted in Figure 5.

The measured and computed RSRs for the BP470, BP540, and the BP635 each agree quite well. The measured and computed RSRs for the BP735 camera, on the other hand, do not agree. We remeasured the RSR of the BP735 camera using a second monochromator to rule out experimental error. The second set of monochromator measurements agree with the measurements of the first. The BP735 filter is manufactured with a bandpass tolerance of ±10 nm, which is consistent with the shift that we observe in our measurements. Comparing the measured RSRs with a calculation is useful, but the measured RSRs are a more reliable estimation of each sensor’s behavior and we use them to calibrate the system.

### 3.5. Calibration

We used the integrating sphere and stable plasma light sources to create a uniform radiance field. Importantly, the lights were connected to the integrating sphere with a variable aperture that allowed us to modulate the spectral radiance intensity. And since each pixel responds in effectively the same way to an incident radiance, a single image samples the normal distribution of digital numbers the camera might record at each brightness level. The absolutely calibrated spectrometer (i.e., calibrated against a NIST-traceable source) with 1% measurement uncertainty in the VIS-NIR measures the spectral radiance of the integrating sphere.

**Figure 6:**
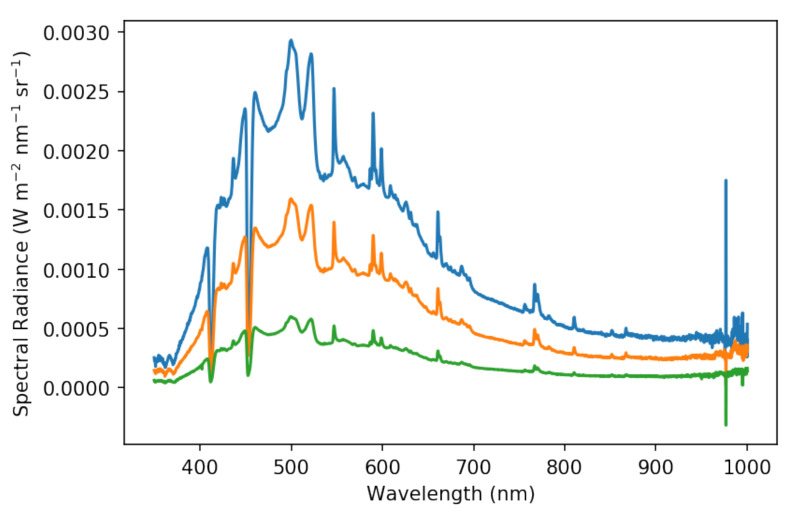
The spectral radiances, acquired by the absolutely calibrated spectrometer, used to sample the dynamic range of our BP540 camera as an example. The absolute intensity of the spectral radiances used to calibrate each camera vary, but the shape remains the same. The spectral radiances need to be folded through the relative spectral response of each camera to obtain effective radiances that are useful for calibration.

The cameras, focused to infinity, were placed in front of the 8-inch aperture of the integrating sphere at a distance of 4.75 inches in order to fill the 67∘ diameter diagonal FOV of the cameras with uniform light. A camera image and a spectrum were gathered simultaneously for three light levels for each camera. Figure 6 shows the range of spectral radiances used for the BP540 camera as an example. The light levels we used were selected based on the distribution of reported digital numbers at each level. Due to vignetting, the cameras recorded a wide range of digital numbers regardless of incident light level. A high light level was selected such that no normally behaving pixel was saturated. Inversely, a low light level was selected such that no normally behaving pixel recorded a digital number lower than the black level we fixed (100 ADU). A middle point was also recorded. The cameras’ linearity, which deviates by only ±0.2%, is measured and reported in Allied Vision’s EMVA 1288 document. With the camera linearity known, three light levels is sufficient.

**Figure 7:**
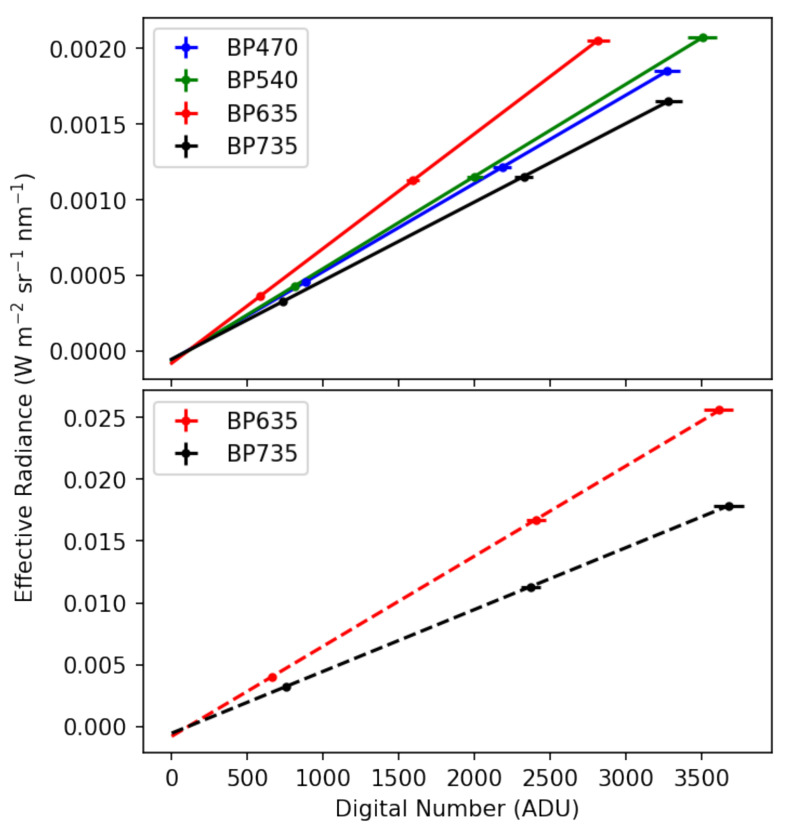
Calibration for each of the 4 cameras at 20 ms exposure time (**top**) and calibration for the BP635 and BP735 cameras at 2 ms of exposure time (**bottom**). The vertical axis is the calculated effective radiance reaching the camera based on the measured radiance spectra and measured relative spectral response of the camera. The fit coefficients and their confidence intervals are reported in Table 1.

Given the RSRs measured in the previous section and Equation (2), we calculated effective radiances and plotted them as a function of average digital number at each light level. The measurement uncertainty in the spectrometer is a function of wavelength, but in the VIS-NIR, the uncertainty is about 1% while the sensors report a digital number with about 2.7% variation. We calibrated using orthogonal distance regressions and each calibration is shown in Figure 7. The regression gives the calibration coefficients *G* and *D* and their associated uncertainties. The calibration coefficients and intercepts for each camera are reported in Table 1. Given the factor of 10 difference between the two exposure times at which these cameras were calibrated, the roughly factor of 10 difference between the coefficients *G* is expected.

### 3.6. Dynamic Range

With known calibration coefficients, we can determine a physically relevant dynamic range for the sensors. The sensitivity ceiling is the maximum recordable ADU value converted to an effective radiance according to Equation (2). The EMVA1288 standard [20] analysis [16] of the Alvium U-1800 319m we use shows that the cameras can reach a maximum of 11.9 bits before the pixel response becomes nonlinear and our calibration coefficients no longer apply. Substituting $N=2^{11.9}-1$ into Equation (2) for each set of calibration coefficients returns the upper bounds reported in Table 2. The sensitivity floor requires a little more effort. The sensitivity floor is determined in part by the intrinsic statistical variability of the sensor’s dark behavior, which we can quantify by examining the average of many dark frames. Our determination of the standard deviation in units of ADU of the average of many dark frames, σ, comes from 100 dark frames. We determined, in Section 3.3, that the statistical behavior of the individual pixels is largely uniform. This means that the standard deviation of the average dark frame is sufficient.

We define here a statistically significant detection when a pixel value exceeds five standard deviations above the camera’s dark behavior. A 5σ threshold may seem steep, but since we have 1544 × 2064 pixels, statistically in each image we should expect about 1 pixel value to rise above the sensitivity floor from noise alone. A looser threshold, take 3σ as an example, implies that about 500 pixels would rise above the sensitivity floor simply due to noise. According to the calibration coefficients, the minimum effective radiance is recorded when N>D, and so a statistically significant detection occurs when N−D≥5σ. Each σ and floor are also reported in Table 2.

Note that the floor and ceiling reported here are valid before accounting for vignetting. Since the vignette of our cameras drops signal by about 35% from center to edge, it is possible for the camera to observe a bright light source near the edges of the image and record a digital number that lies below $2^{11.9}$ bits, which is a valid detection. Correcting for vignetting by dividing by the normalized flat frame boosts that number above the ceilings quoted in Table 2 while remaining a good measurement. The same is true for the sensitivity floor, depending on whether or not pixels near the optical axis fluctuate above the value of the pixel at the optical axis, which would result in dividing by a number larger than one. It is possible that this may drop a significant measurement below the sensitivity floor.

We choose to operate the cameras in 12-bit mode, which corresponds to $2^{12}$ possible ADU bins for signal to be sorted into rather than the 8-bit mode corresponding to $2^{8}$ bins. While this does not increase the dynamic range of the camera (the total range between saturation and the noise floor of a camera at a given configuration), a higher precision increases the value of elevated data products. The ability for the camera electronics to reliably sort a signal into the correct digital number bin depends on both the size of the bin, referred to as the level of digitization, and the camera’s noise. If the bin size is too small in comparison with the expected noise, then noise dominates the camera’s sensitivity. We can quantify this by taking the ratio of the bit depth to σ, also reported in Table 2.

Our cameras’ sensitivity is superior to 8-bits of depth across the board, while for some cameras and configurations we find the noise tends to dominate. Given the criterion for linear response up to $N=2^{11.9}-1=3821$ reported in the EMVA1288 analysis document [16], the camera sensitivity is sufficient for our application.

### 3.7. Effective Radiance Uncertainty

Quantifying the radiance measurement uncertainty of our sensors requires calculating the uncertainty ΔL from Equation (2) in the standard way, which is appropriate because *N*, *G*, and *D* are not correlated:

(4) $$\Delta L_{\mathrm{eff}}\left(G,N,D\right)=\sqrt{{\left(\frac{\partial L_{\mathrm{eff}}}{\partial G}\Delta G\right)}^{2}+{\left(\frac{\partial L_{\mathrm{eff}}}{\partial N}\Delta N\right)}^{2}+{\left(\frac{\partial L_{\mathrm{eff}}}{\partial D}\Delta D\right)}^{2}}.$$

**Figure 8:**
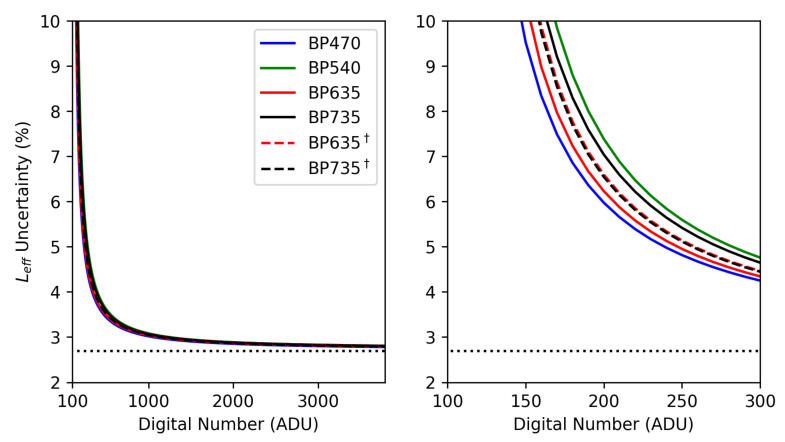
Percent uncertainties calculated by taking the ratio of Equations (2) and (4). The short-exposure calibrations are marked with a dagger. The uncertainty asymptotes near N=100 ADU because each *D* is near 100 ADU. The measurement uncertainty approaches 2.7%, plotted with a horizontal dotted line, because the CMOS sensor measurement uncertainty dominates as *N* increases.

The slope and intercept confidences, ΔG and ΔD, respectively, are reported in Table 1. The digital number uncertainty ΔN is proportional to *N*, which means the uncertainty calculated above is a function of the digital number measured. We perform the calculation above, assume ΔN=0.027·N, and plot percent uncertainties ΔL/L as a function of *N* in Figure 8.

Naturally, there is a singularity near N=100 ADU, which is easily seen when comparing the calibration coefficients *D* with N≈100 ADU in the context of Equation (2). Given that ΔG and ΔD are constants and that ΔN is proportional to *N*, the percent error should approach the CMOS measurement uncertainty, 2.7%, as is indeed seen.

## 4. Data Product Demonstration

**Figure 9:**
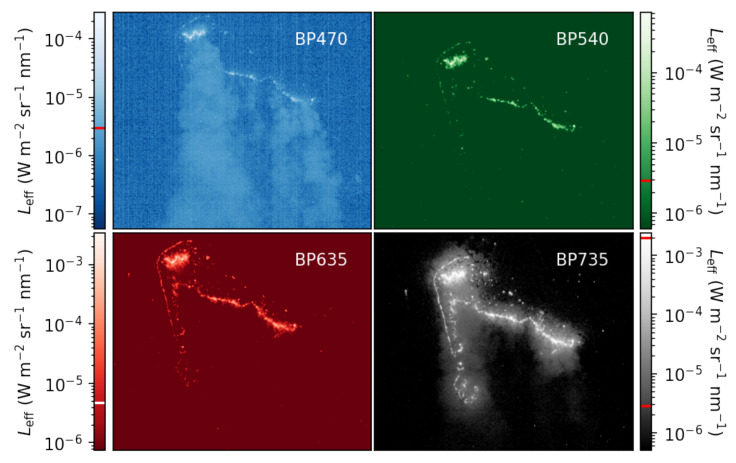
Calibrated images in false color of an agricultural burn. Horizontal lines on the color bars indicate each camera’s sensitivity floor, with a second line on the BP735 image that indicates the ceiling. The images were treated with a flat field, so the floor and ceiling are here as a reference. The images were acquired near (ϕ,λ)=(31.7454,−82.0087) on 9 February 2024 at 5:40 PM. The images were taken at an altitude of 1979 m above sea level, or about 1930 m above ground level, which corresponds to a pixel size of about 0.83 m, which implies the 672 × 782-pixel scene here represents about 0.36 square kilometers. Sunset occurred at 6:17 PM. With a low amount of daylight, some smoke is visible in the blue channel from the fire and the fire is still resolved in the near-infrared channel, where solar contamination is strongest.

As an example, we show calibrated images of nighttime light observations, on a log scale, of an agricultural burn in Figure 9, with the acquisition details outlined in the figure caption. The images were calibrated using Equation (2) and then divided by master flat frames with optical axes found according to Section 3.2 to correct for vignetting. We will conduct a campaign in the future that will allow us to perform a comprehensive analysis of our data products. We are limited in a large part by the lack of a georectification pipeline, which is required to compare data across sensors. Georectification for this iteration of NightHawk is underway and will be presented in a later manuscript led by S. Tammes.

We expect most signal from the fire itself to appear in the BP735 image, which is borne out in the data. Decreasing levels of signal with decreasing wavelength are also expected, which is visually apparent when we compare the shape, size, and continuity of the fire fronts between the images in the set, which is especially true in the BP540 image. In the BP470 image, we are also able to discern the smoke column with acceptable signal, since the majority of the smoke plume is above the sensitivity floor of the BP470 camera. The ability to observe smoke with NightHawk enables additional science beyond detecting and characterizing burning biomass flaming. The light reflecting off of the smoke in this set of images is likely scattered light from the sun at dusk.

Light in the VIS-NIR is attenuated as it travels toward the sensor and through smoke. Given that attenuation of VIS-NIR light by smoke is inversely proportional to wavelength, light will be most attenuated in the BP470 band and least attenuated in the BP735 band. This does not confound our sensor’s ability to identify natural flaming since, in Kaaret et al. [15], we demonstrated that the key band ratio for identifying flaming is BP735/BP635 (burning biomass has the largest ratio in comparison with other nighttime light sources). Light in the BP635 bandpass will be more attenuated than light in the BP735 bandpass, which enhances the flaming detection criterion.

**Figure 10:**
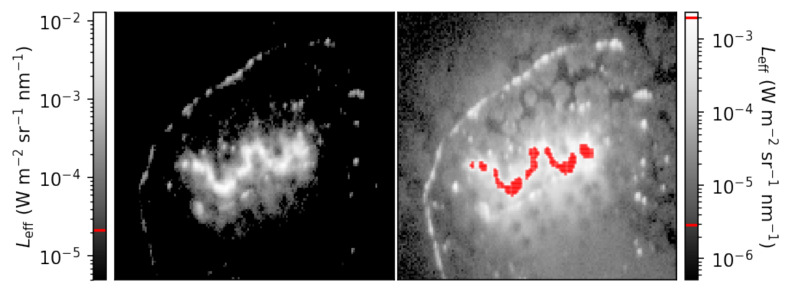
A closer view of the brightest portion of the fire in Figure 9. The short-exposure image (**left**) has no data above its sensitivity ceiling. The long-exposure section on the (**right**) contains 239 pixels, highlighted in red, that are above the sensitivity ceiling of the camera operating at 20 ms of exposure time.

The majority of pixels in each image are above each sensitivity floor, due in part to the presence of sunlight, with no saturation in the BP470, BP540, and BP635 images. There are 260 pixels that are saturated in the BP735 image, with an additional 30 pixels that are above the sensitivity ceiling of the camera operating at 20 ms (detected prior to correcting for vignetting). There is a companion observation at 2 ms of exposure time, which we can use to verify that our strategy of using multiple exposure times is effective. We compare the data in the BP735 image at 20 ms of exposure time with the data in the 2 ms exposure in Figure 10.

The short-exposure companion observation contains no pixels that report values above the camera’s sensitivity ceiling. It is also clear that, for this set of images, operating at 2 ms of exposure time is too short to observe much reflected NIR light in comparison with observable structures in the 20 ms image. It is difficult to immediately integrate data from both images. First, we require georectification to tell us which pixel corresponds to what location on the ground. Second, even though both exposure times are calibrated with equal fidelity, the two exposure times have different ground sample sizes. Both of these problems require careful consideration of the attitude, velocity, and altitude data measured by the IMU. An appropriate georectification pipeline can coordinate the two companion images such that oversaturated pixels in the long-exposure image and undersaturated pixels in the short-exposure image can be unified, as well as integrating information across sensors.

## 5. Summary

Nighttime light observations of wildfire activity are increasingly important with increasing nighttime wildfire activity across the globe [14]. Our device observes VIS-NIR light at night which, when combined with our filter suite, allows us to target signal from active flaming. In this manuscript, we showed the following:

- When radiometrically calibrated, NightHawk has effective radiance sensitivity that spans between $10^{-6}$ and $10^{-3}$ W m^−2^ sr^−1^ nm^−1^ with the cameras calibrated at 20 ms of exposure and $10^{-5}$ and $10^{-2}$ W m^−2^ sr^−1^ nm^−1^ at 2 ms of exposure, with uncertainties below 5% across the board (Table 1).
- The range of the sensitivity floor, depending on the filter, is 3–5 $10^{-6}$ W m^−2^ sr^−1^ nm^−1^, and the sensors have dynamic ranges between 2946 and 4222.
- Upon deployment, NightHawk can indeed observe and quantify NTL from burning biomass at scale, including resolving fire fronts.

The NightHawk platform prototype as it stands democratizes high-quality NTL observations of natural flaming, and provides a proof of concept for a simpler, broadband multispectral regime for remotely sensing burning biomass. This prototype also demonstrates the VIS-NIR technology of a remote sensing platform suitable for flight on a manned aircraft or UAV that is optimized to measure the VIS-NIR portion of burning biomass’s visible emission fraction. If paired with a suitable IR sensor, remotely sensing the combustion efficiency of natural flaming is achievable [5,6].

## Figures and Tables

**Table 1:** Calibration coefficients obtained by an orthogonal distance regression for each camera at 20 ms and 2 ms^†^ of exposure time. The calibration assumes 1% uncertainty in the effective radiance measurement (appropriate in the VIS-NIR) and 2.7% uncertainty in the corresponding digital number recorded by the camera. The calibration analysis does not include a dark subtraction, so the coefficient *D* should closely resemble the dark level (fixed at 100 ADU) of the camera.

Camera	*G*	D
	$10^{-7}$ **(W m^−2^ **sr**^−1^ nm^−1^ ADU^−1^)**	**(ADU)**
BP470	5.827 ±0.012	98.9 ±2.7
BP540	6.088 ±0.023	104.7 ±4.5
BP635	7.571 ±0.022	104.4 ±2.5
BP735	5.186 ±0.021	100.9 ±4.4
BP635 ^†^	72.84 ± 0.20	108.4 ± 2.7
BP735 ^†^	49.99 ± 0.12	107.6 ± 2.7

**Table 2:** The standard deviation, sensitivity floor, ceiling, and dynamic range of each camera at 20 ms and 2 ms ^†^ of exposure time.

Camera	σ	Floor	Ceiling	Range
	**(ADU)**	$10^{-6}$ **(W m^−2^** **sr** **^−1^** **nm** **^−1^** **)**	$10^{-3}$ **(W m^−2^** **sr** ^−1^ **nm** ^−1^ **)**	
BP470	1.03	2.99	2.23	3976
BP540	0.97	2.95	2.33	4222
BP635	1.26	4.75	2.89	3250
BP735	1.10	2.86	1.98	3723
BP635 ^†^	1.39	50.56	27.83	2946
BP735 ^†^	0.86	21.58	19.10	4762

## Data Availability

Dataset available on request from the authors.

## Abbreviations

The following abbreviations are used in this manuscript:

NTL	Nighttime light
SWIR	Short-wave infrared
MWIR	Mid-wave infrared
FRP	Fire radiative power
MCE	Modified combustion efficiency
VIIRS	Visible Infrared Imaging Radiometer Suite
VIS-NIR	Visible to near-infrared
VEF	Visible emission fraction
ADU	Analog-to-digital units
FOV	Field of view
RSR	Relative spectral response
